# Cytological Spectrum of Pulmonary Histoplasmosis Diagnosed by Bronchoalveolar Lavage: 12 Years of Experience in French Guiana

**DOI:** 10.3390/jof7070576

**Published:** 2021-07-19

**Authors:** Kinan Drak Alsibai, Houari Aissaoui, Antoine Adenis, Morgane Bourne-Watrin, Felix Djossou, Loïc Epelboin, Denis Blanchet, Magalie Demar, Pierre Couppié, Mathieu Nacher

**Affiliations:** 1 Department of Pathology, Centre Hospitalier de Cayenne Andrée Rosemon, 97300 Guiana, France; 2 Center of Biological Resource (CRB Amazonie), Centre Hospitalier de Cayenne Andrée Rosemon, 97300 Guiana, France; 3 Department of Medicine, Pulmonology Unit, Centre Hospitalier de Cayenne Andrée Rosemon, 97300 Guiana, France; houari.aissaoui@ch-cayenne.fr; 4 Centre d’Investigation Clinique «Inserm CIC 1424», Centre Hospitalier de Cayenne Andrée Rosemon, 97300 Guiana, France; antoine.adenis@ch-cayenne.fr (A.A.); mathieu.nacher66@gmail.com (M.N.); 5 Department of Dermatology, Centre Hospitalier de Cayenne Andrée Rosemon, 97300 Guiana, France; mbournewatrin@yahoo.fr (M.B.-W.); pierre.couppie@ch-cayenne.fr (P.C.); 6 Unité des Maladies Infectieuses et Tropicales, Centre Hospitalier de Cayenne Andrée Rosemon, 97300 Guiana, France; felix.djossou@ch-cayenne.fr (F.D.); loic.epelboin@ch-cayenne.fr (L.E.); 7 Laboratory of Parasitology and Mycology, Centre Hospitalier de Cayenne Andrée Rosemon, 97300 Guiana, France; denis.blanchet@ch-cayenne.fr (D.B.); magalie.demar@ch-cayenne.fr (M.D.)

**Keywords:** bronchoalveolar lavage, histoplasmosis, cytopathology, histoplasma capsulatum, clustering pattern

## Abstract

Disseminated histoplasmosis is a major cause of mortality in HIV-infected patients. Rapid and efficient diagnosis of *Histoplasma capsulatum* is crucial. Cytopathology is available in most hospitals and represents a rapid diagnostic alternative. In this study, we reviewed 12 years of experience to describe the cytology of histoplasmosis diagnosed by bronchoalveolar lavage (BAL) in relation to patient characteristics. BAL-diagnosed pulmonary histoplasmosis concerned 17 patients (14 HIV+). BAL cellularity ranged from 76,000 to 125,000 cells/mL in HIV patients, and 117,000 to 160,000 cells/mL in non-HIV patients. Macrophages predominated in all HIV patients (from 60% to 88%), lymphocytic infiltrates ranged from 5% to 15%, and neutrophils were very heterogeneous (from 2% to 32%). The number of *H. capsulatum* at hot spots seemed greater in HIV-infected than in immunocompetent patients (9 to 375 vs. 4 to 10) and were inversely proportional to the CD4 counts. Yeasts were both intracellular and extracellular in 85.7% of the HIV patients. This is the most comprehensive series detailing the cytological aspects of BAL in the diagnosis of *H. capsulatum*, focusing on the number of yeasts and their clustering pattern. The cytological examination of the Gomori-Grocott-stained BAL allows a reliable diagnosis of histoplasmosis.

## 1. Introduction

**Figure 1:**
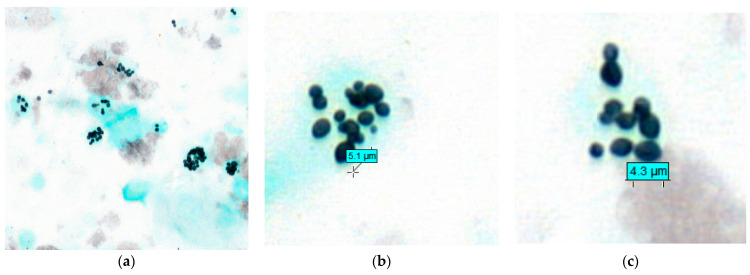
Bronchoalveolar lavage showing *Histoplasma capsulatum*. (**a**) Gomori-Grocott stain at ×40. *H. capsulatum* appears as small spherical or ovoid yeasts <6 µm in size. After inhalation, it makes a dimorphic transition to yeast, enters macrophages, and is then able to proliferate and survive intracellularly, and sometimes extracellularly. (**b**,**c**) Gomori-Grocott stain at ×100 and ×120, respectively.

*Histoplasma capsulatum* (*H. capsulatum*) is the causative agent of histoplasmosis. In organs, *H. capsulatum* appears as small spherical or ovoid yeasts measuring between 2 and 6 µm [1]. After inhalation, it makes a dimorphic transition to yeast to enter the hosts macrophages. Yeasts are able to proliferate and survive intracellularly; they may persist during asymptomatic infection and then reactivate or proliferate during active infection [2] (Figure 1). The severity of histoplasmosis is influenced by the intensity of exposure to yeasts and to the host’s immunity. Thus, there is a spectrum of severity ranging from asymptomatic infection to mild lung disease in the case of normal immunity, whereas a high inoculum can result in severe lung infections regardless of a patient’s past medical history. Disseminated histoplasmosis is considered a major but often neglected infection in patients with acquired immunodeficiency syndrome (AIDS) [3,4,5]. In Latin America, histoplasmosis is often classified as the most common cause of death in patients with advanced HIV disease (defined as a cluster of differentiation-4 (CD4) T-cell count below 200 cells/mm^3^, or a WHO clinical stage 3 or 4 event at presentation for care [6,7]. 

French Guiana, a European territory in Latin America, is endemic for histoplasmosis and has a high prevalence of HIV infection [5,8]. In French Guiana, the incidence of disseminated histoplasmosis in HIV patients is estimated to be, overall, 1.5 per 100 persons/year. This incidence rate is even higher in HIV patients with CD4 counts below 50 cells/mm^3^ (>10 per 100 persons/years) [9,10]. 

The reference methods for detecting *H. capsulatum* in immunocompromised patients are fungal cultures performed by laboratories and cytological and/or histological examination performed in pathology departments with a sensitivity of 80% and 65%, respectively [10,11,12]. Nevertheless, given the complexity of a pathologist’s specialty, the sensitivity of cytological and histological examination in the diagnosis of histoplasmosis depends on the pathologist’s experience in the detection of pathogens [12]. 

Considering the limited distribution and availability of antigen detection tests in daily practice outside of the United States, molecular detection by polymerase chain reaction (PCR) and serological detection in plasma are used elsewhere. However, in low- and middle-income countries molecular diagnosis is mostly unavailable [8,11,13,14]. In addition, the performance of diagnostic methods depends on the type and quality of specimen, the clinical pattern and tissue sampling, and the immune status of the patient [13,15]. Cytological and histological examination is usually present in most hospitals and can represent an important diagnostic alternative [12]. 

Bronchoalveolar lavage (BAL) is a very cost-effective and relatively invasive examination for the investigation of lung disease in immunocompromised patients. It can be performed in most patients and allows for the differential diagnosis between bacterial, viral, parasitic, or fungal lung infections as well as for non-infectious pathologies. BAL fluid is also a good technique for exploring the pulmonary inflammatory microenvironment. BAL allows for the determination of the type of immune/inflammatory cells, their size and shape, and their content. BAL is also used to perform cell counting and to search for pathogens. Thus, BAL is an important tool in the diagnosis of inflammatory, autoimmune and infectious pulmonary diseases [16]. Hence, in patients with advanced HIV, it has been a major diagnostic procedure to identify infecting pathogens. Although over 40 years have passed since the first description of AIDS, there have been few detailed descriptions of the particularities of BAL in histoplasmosis [17,18,19].

In this study, we reviewed 12 years of experience in our Department of Pathology (2008–2020) for the diagnosis of pulmonary histoplasmosis from the BAL fluid. We describe for the first time the inflammatory microenvironment of BAL in the case of *H. capsulatum* infection and its possible relation to a patient’s immune status.

## 2. Ethical Statement

Human immunodeficiency virus (HIV) and histoplasmosis co-infections were included in the French Guiana HIV and Histoplasmosis Database. Inclusion criteria were: confirmed HIV infection, age > 18 years, and proven first episode of histoplasmosis (European Organization for Research and Treatment of Cancer/Mycosis Study Group (EORTC/MSG) criteria) [20]. This database included incident cases of HIV-associated histoplasmosis in French Guiana. Clinical, biological, immunovirological and therapeutic data were collected in a standardized form. Hospitalized cases of HIV-associated histoplasmosis were included. Furthermore, all patients in our institute were informed that their biological samples and associated data could be used for scientific purposes, and that they had the right to object in compliance with the European data protection regulation (GDRP 2018). The research in this database has been approved by the Ethical Evaluation Committee of the National Institute for Health and Medical Research (IRB0000388, FWA00005831 18 May 2010), the Consultative Committee on Information Processing for Health Research (CCTIRS) (No. 10.175 bis, 10 June 2010), and the French National Commission for Information Technology and Civil Liberties (CNIL) (No. JZU0061856X, 16 July 2010).

## 3. Patients and Methods

A retrospective single-center study was performed between January 2008 and December 2020 in the Department of Pathology at the Cayenne Hospital Center in patients with proven pulmonary localization of histoplasmosis diagnosed by BAL according to the EORTC/MSG criteria (histopathological analysis, direct microscopy, or cultures of specimens obtained from an affected site showing the *H. capsulatum* yeasts) [20]. Cayenne Hospital is the largest public hospital in French Guiana and is the reference center for tropical infectious diseases and for the management of HIV-infected patients.

All cases were reviewed by an expert cytopathologist. Data regarding age, sex, and immune status (HIV and non-HIV) were collected as well as the clinical pattern (pulmonary only or extrapulmonary-disseminated), and the CD4 and CD8 counts in the diagnosis of HIV patients. 

In non-smoking adults, a BAL is considered within normal limits when the cellularity is approximately 150,000 cells/mL with a cellular formula: macrophages between 80% and 90%, lymphocytes between 5% and 10%, neutrophils at <5%, and eosinophils at <2%. In practice, a percentage of lymphocytes of >20% is considered as hyperlymphocytosis and should alert the cytopathologist to a chronic infection, an autoimmune disease, or a lymphoma. In this case, lymphocyte typing can be performed from the BAL fluid.

In this study, the total cell count was performed on non-centrifuged BAL using a Malassez hemocytometer (cells/mL). To facilitate the interpretation of BAL cellularity, we rounded the last three digits of the cellularity to 0 when the count was less than 100,000 cells/mL (e.g., 76,300 becomes 76,000 cells/mL), and the last four digits when the count was greater than 100,000 cells/mL (e.g., 161,400 becomes 160,000 cells/mL).

After centrifugation, all BALs were stained with the May-Grünwald Giemsa (MGG) stain to assess cell counts. The Papanicolaou stain was also available in 4 cases. The special stain Gomori-Grocott was routinely performed in all BALs. 

We reinterpreted the BALs by specifying the number of cells and the percentage of the four types of inflammatory cells: macrophages, lymphocytes, neutrophils, and eosinophils. We counted a total of 500 inflammatory cells per slide in at least 3 different fields with a light microscope at a ×40 objective. The presence of some inflammatory elements was exceptional (<1%), so we excluded them from the cell formula stating that their presence was rare. 

The Golde score was used to assess alveolar hemorrhage by calculating the number of hemosiderin-laden macrophages and the intensity of iron loading by Perls stain. The Golde score requires counting 100 macrophages and assigning a value from 0 to 4, based on the density of hemosiderin in their cytoplasm (Golde score: 0 to 20 normal, 20 to 70 intermediate resorption, >70 high resorption, and >100 occult alveolar hemorrhage).

Since the ×40 objective of the light microscope (×400 magnification) is the one most used by pathologists in daily practice, we calculated the number of *H. capsulatum* (Gomori-Grocott positive yeasts) per field where they were most numerous (called a hot spot). We identified the main location of the *H. capsulatum* on the cytological slides of BAL. We classified this location based on three categories: intracellular (IC: yeasts located mainly in the cytoplasm of macrophages), extracellular (EC: free yeasts outside macrophages), or IC + EC (IC with a significant number of EC yeasts). In some cases, it was difficult to assess the location of yeasts by the Gomori-Grocott stain alone. In this case, the interpretation was performed with the MGG stain.

We also described the clustering pattern of the *H. capsulatum* yeasts. We classified these clustering patterns into three groups: *type I*: isolated yeasts and/or small clusters or small chains of <5 yeasts; *type II*: I + clusters or chains of >5 yeasts; type *III*: II + rosettes. The rosettes pattern corresponds to round structures made up of one or more layers of yeasts (often > 10 yeasts) leaving a pseudo-cavity in the center.

STATA© (College Station, TX, USA) was used for the statistical analysis. The medians of BAL cellularity (Malassez, cells/mL) and the number of yeasts per hot spot (×40) among HIV and non-HIV patients with the interquartile range (IQR: 25% to 75%) were calculated and compared using the Mann–Whitney test. The relationship between the CD4 count level and the number of *H. capsulatum* yeasts per hot spot among HIV patients was explored using the Spearman Rank Correlation coefficient (Spearman Rho). Statistical significance was set at *p* < 0.05.

## 4. Results

We diagnosed pulmonary histoplasmosis by cytological examination of BALs in 17 patients (12 males and 5 females); M:F sex ratio 2.4:1 and aged 25 to 65 years, including 14 HIV patients and three non-HIV patients. The delay of the BAL results was between 2 and 5 days. CD4 counts in HIV patients ranged from 6 to 227 cells/mm^3^ at the time of diagnosis. Twelve HIV patients (12/14: 85.7%) had advanced HIV disease with a CD4 count of <200 cells/mm^3^, and eight patients (8/12: 66.7%) had a CD4 count of <50 cells/mm^3^ (Table 1). Eight HIV patients had disseminated histoplasmosis (8/14: 57.1%). All eight patients had a CD4 count of <200 cells/mm^3^, five of them had a CD4 count of <50 cells/mm^3^.

The immune status of the three non-HIV patients was confirmed by a negative HIV serological test. One of these patients who was 65 years old was under immunosuppressive therapy following renal transplantation 7 years earlier due to diabetic nephropathy. This patient consulted the emergency unit of our institute following a fever with asthenia. The chest CT scan showed a diffuse micronodular image in both lungs. The youngest non-HIV patient was 36 years old and had consulted the emergency unit because of an altered general condition with fever. The chest CT scan showed *miliary* opacities in both lungs with multiple lymphadenopathy. The last non-HIV patient presented with an excavated consolidation of the upper lobe of the left lung. 

The cellularity of BAL ranged from 76,000 to 125,000 cells/mL in HIV patients, and from 117,000 to 160,000 cells/mL in non-HIV patients (Table 1). The median of BAL cellularity was 91,000 cells/mL (IQR 25–75: 83,000–100,000) among HIV patients, and 138,000 cells/mL (117,000–160,000) in non-HIV patients (*p* = 0.0166). The cellular formula of BAL was predominantly macrophages in all HIV patients (macrophages from 60% to 88%), and also in the three non-HIV patients (macrophages: 59%, 73%, and 82%). We found a mild lymphocytic infiltrate ranging from 5% to 15% in HIV patients and from 12% to 18% in non-HIV patients. No significant hyperlyphmocytosis (>20%) was detected. The presence of neutrophils was slightly higher in all patients, but with a very heterogeneous distribution (from 2% to 32%). The neutrophils were higher than normal (≥5%) in the majority of HIV patients (11/13 cases: 84.6%) and in the three non-HIV patients (100%). The presence of eosinophils remained exceptional (0% to 2%) in all patients (Table 1). We found no evidence of alveolar hemorrhage, and the Golde score was often within normal limits (between 0 and 20) in almost all HIV and non-HIV patients (16/17 cases: 94%). Only one HIV patient had a Golde score of 50, which was in favor of an intermediate resorption process. 

**Figure 2:**
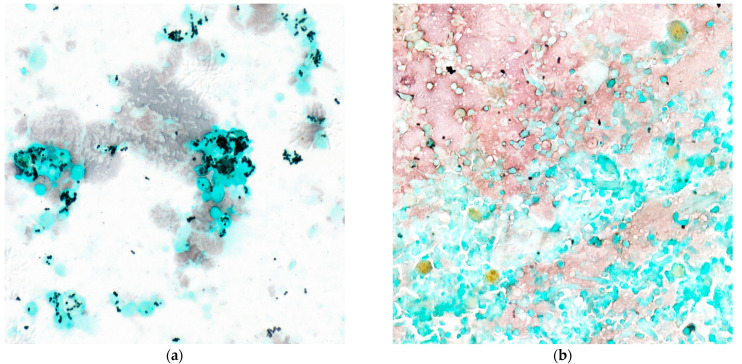
The number of *Histoplasma capsulatum* by hot spots with the Gomori-Grocott stain (light microscopy at ×40). (**a**) Shows numerous intracellular and extracellular yeasts by hot spots in a patient with a CD4 count of <200 cells/mm^3^. (**b**) Shows a few isolated yeasts sometimes arranged in small clusters.

We confirm in this series the old finding describing *H. capsulatum* var. *capsulatum* yeasts as round or ovoid in shape and measuring from 2 to ≤6 μm (Figure 1). The number of *H. capsulatum* per hot spot was significantly higher in HIV patients (9 to 375/hot spot) (Figure 2). The number of *H. capsulatum* per hot spot remained low to moderate in non-HIV and non-immunocompromised patients (4 and 10/hot spot). The median count of *H. capsulatum* per hot spot was 91.5 (5–133) among HIV-infected patients and 10 (4–66) in non-HIV median (*p* = 0.043). There was a negative but statistically non-significant correlation between the CD4 count and the number of *H. capsulatum* yeasts among the HIV patients (*n* = 14) shown by a Spearman Rho of −0.09 (*p* = 0.74) (Scatter plot).

The *H. capsulatum* yeasts were at least IC in all HIV and non-HIV patients. Yeasts were only IC in the three non-HIV patients, and in only two HIV patients (14.4%) with *H. capsulatum* counts ≤ 25/hot spot. In contrast, yeast was both IC and EC in HIV patients with higher yeast counts (>25/hot spot).

**Figure 3:**
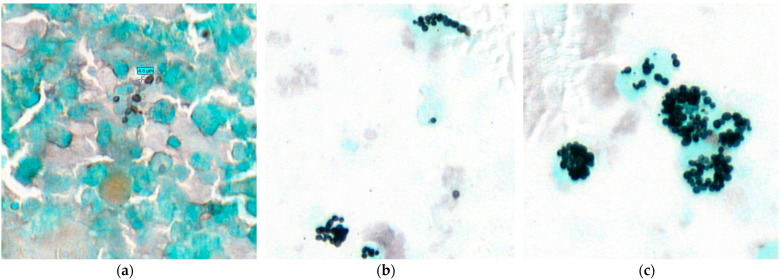
Clustering pattern of *Histoplasma capsulatum* yeasts in the bronchoalveolar lavage. We could identify three clustering patterns. Type I: isolated yeasts and/or small clusters or small chains of <5 yeasts (**a**) Gomori-Grocott stain at ×60. Type II: type I + clusters or chains of >5 yeasts, (**b**) Gomori-Grocott stain at ×40. Type III: type II + rosettes structures. The rosettes pattern corresponds to round structures made of one or more layers of yeasts (often > 10 yeasts) leaving a pseudo-cavity in the center (**c**) Gomori-Grocott stain at ×60.

In HIV patients, the yeast clustering pattern was type I in 4/14 cases (28.6%), type II in 7/14 cases (50%) and type III in 3/14 cases (21.4%) (Figure 3). Among the non-HIV patients, two patients had a type I clustering pattern, and only one patient had a type II pattern. No patient had a rosette-type pattern.

We found that in all eight patients with disseminated histoplasmosis, *H. capsulatum* yeasts were both intra- and extracellular. The yeasts had a type II or type III clustering pattern. The number of yeasts varied between 78 and 375 per hot spot. 

Concerning non-HIV patients, the patient under immunosuppressive drugs following a renal transplantation had the highest number of yeasts (66/hot spot), with a type II clustering pattern.

A coinfection with *Pneumocystis jirovecii* was detected by BAL in two cases and with *Mycobacterium tuberculosis* in one case, all in HIV patients. *Cytomegalovirus* (CMV) infection was detected by PCR on BAL in the renal transplant patient.

## 5. Discussion

To our knowledge, this is the most comprehensive series in the literature detailing the cytological aspects, cellularity, and cell formula of BAL in the diagnosis of *H. capsulatum*, with particular attention to the number of yeasts and their clustering pattern. In HIV-infected patients, all BALs were macrophage-dominant. In a recent experiment concerning the histological appearance of histoplasmosis, we demonstrated that the macrophage-predominant microenvironment is the most frequent in HIV patients [12]. This microenvironment is represented by two histological forms: the granulomatous (or tuberculoid) form and the intermediate form. These two forms are rich in macrophages or epithelioid cells derived from macrophages. In addition, in the anergic form and the sequelae form, the host inflammatory responses to *H. capsulatum* are very weak [12]. Nevertheless, it should be remembered that BAL is a medium naturally rich in alveolar macrophages. Therefore, the cellularity of a BAL must be significantly increased before concluding that the number of macrophages is pathological. Given these data, we can speculate that when a BAL in an HIV-infected patient co-infected with *H. capsulatum* has a cellularity >150,000 cells/mL, it may correspond to a granulomatous or intermediate form of histoplasmosis. On the other hand, when a BAL has a significantly lower than average cellularity (<100,000 cells/mL), it may correspond to anergic or sequelae histoplasmosis. Nevertheless, hypothesizing a correlation between the cytological and histological appearance cannot be formally confirmed in this cohort, because there was no indication to perform a lung biopsy after the diagnosis of pulmonary histoplasmosis on BALs. 

**Figure 4:**
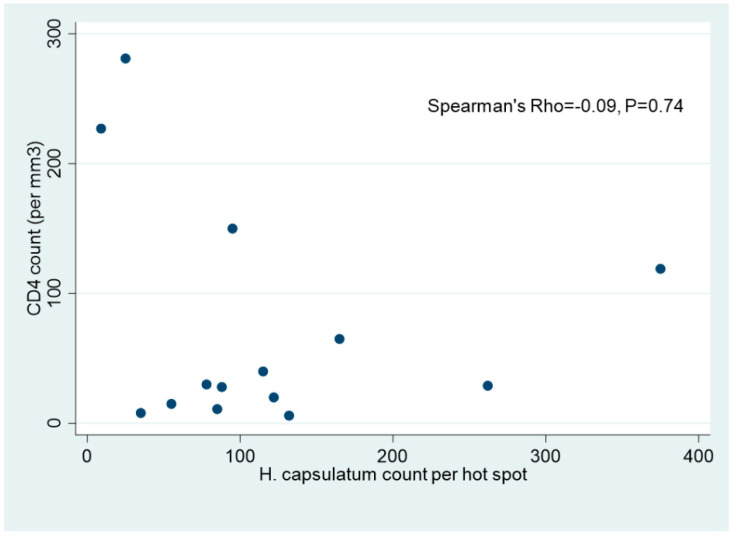
Scatter plot showing a negative but statistically non-significant correlation between the CD4 count/mm^3^ and the number of *H. capsulatum* yeasts per hot spot (×40) among HIV patients (*n* = 14) with a Spearman Rho of −0.09 (*p* = 0.74).

We found a non-significant trend between the increased number of *H. capsulatum* per hot spot and the low CD4 count in HIV patients (Figure 4). Patients who had a CD4 count of <200 cells/mm^3^ at the time of diagnosis (12/14 patients; 85.7%) often had a high number of *H. capsulatum* (35 to 375/hot spot). In addition, among the non-HIV patients, the immunosuppressed patient had the highest number of yeasts (66/hot spot). The yeast localization and clustering pattern appeared to be related to the immune status and/or CD4 count at diagnosis. Yeasts were exclusively IC in the two immunocompetent patients and in only two HIV patients (14.4%) whose *H. capsulatum* counts were <25/hot spot. In contrast, yeasts were both IC and EC in HIV patients who had a number of yeasts >25/hot spot (85.6%).

In HIV patients with a CD4 count of <200 cells/mm^3^, the type II clustering pattern was the most frequent (7/14 cases: 50%). In addition, the type III rosette-like pattern was identified in the three HIV patients who had the highest number of yeasts (165, 262 and 375/hot spot). This is probably related to the high proliferation rate of *H. capsulatum* yeasts in the bronchoalveolar space in these patients. 

This study highlights the importance of employing routine special stains in the cytological examination of BAL, especially in HIV and immunocompromised patients. The Gomori-Grocott stain, besides being the standard stain for the diagnosis of fungal filaments, spores, and yeasts plays a role in the diagnosis of *Pneumocystis jirovecii* [21]. The latter is also marked by Grocott’s stain. The specific form of *Pneumocystis* and its mode of clustering as extracellular cysts allows discernment from *H. capsulatum* yeasts. In our department, the use of a *Pneumocystis jirovecii* antibody in immunocytochemistry on a cytological sample of BAL allows for confirmation of the diagnosis. In addition, the Ziehl–Neelsen stain is useful for the identification of acid-fast bacteria, mainly mycobacteria [10].

In previous studies, we have demonstrated the effectiveness of BAL in the diagnosis of pulmonary localization of histoplasmosis [12,22]. Comparing the different diagnostic methods of histoplasmosis in our institute between 1981 and 2014, direct examination and fungal cultures of BAL performed by the Mycology Laboratory were more frequent than cytopathological examination [22]. Overall, fewer than half of the specimens diagnosed as *H. capsulatum* had cytological or histological examination, whereas more than half of the specimens examined by the pathologist were positive [22]. Prior to 2015, within our department, fewer than 10% of these BALs received a request for cytological examination. Cytopathology examinations have a quick turn-around time, similar to the most recent developments in antigen or molecular biology for the diagnosis of histoplasmosis. This is a wasted diagnostic opportunity not to send samples for cytopathology stains in the case of a negative direct mycological examination, as fungal cultures can take several weeks. However, since 2015, we have seen a marked improvement in this practice. Since 2019, BALs have been routinely sent to the Mycology Laboratory as well as to the Department of Pathology [12]. Since then, the MGG, Gomori-Grocott, Ziehl–Neelsen and Perls stains have been performed systematically in our department on all BALs regardless of the immune status of the patient. 

In this series, the HIV patients with disseminated histoplasmosis (i.e., more than one infection site) had a CD4 count of <200 cells/mm^3^, extracellular *H. capsulatum*, >50 yeasts per hot spot, and either cluster pattern II (cluster or chain of >5 yeasts) or III (rosette > 10 yeasts). Hence, we hypothesized that the location, number, and clustering pattern of *H. capsulatum* on microscopical examination of BALs may actually reflect the dissemination of these yeasts and the subsequent severity of the disease. Therefore, in our opinion, these elements should alert the cytopathologist and the medical team of a possible disseminated and potentially severe disease. Nevertheless, this hypothesis should be tested in a larger cohort.

## 6. Conclusions

This is the largest series detailing the cytological aspect of BAL in the diagnosis of *H. capsulatum*, focusing on the number of yeasts and their clustering pattern. The cytological examination of BAL allows a reliable and rapid result of histoplasmosis by using the Gomori-Grocott stain. All BALs had a predominantly macrophage cell count. Lymphocytes and neutrophils were present but in low to moderate numbers. The number of *H. capsulatum* was higher in HIV-infected than in immunocompetent patients and seemed higher when the HIV patients’ CD4 counts were low; however, this failed to reach statistical significance, possibly given the small sample size. In HIV-infected patients, yeasts were both intracellular and extracellular especially when their number was >25 per hot spot (×40). Our data raise the hypothesis that the microscopical pattern of *H. capsulatum* on BAL may be a reflection of disseminated disease and a potential proxy to disease severity from a bench perspective.

## Figures and Tables

**Table 1:** Data from the 17 patients (14 HIV and 3 non-HIV) with pulmonary histoplasmosis. The characteristics of their BALs (cellularity and cell formula) and the *Histoplasma capsulatum* yeasts detected by the Gomori-Grocott stain at a ×40 field (hot spot). HC: *Histoplasma capsulatum.* IC: intracellular; EC: extracellular. Cluster’s pattern: type I: small cluster or chain < 5 yeasts; type II: I + cluster or chain > 5 yeasts; type III: II + rosette.

HIV and Non-HIV Patients						Bronchoalveolar Lavage					Gomori-Grocott Stain		
	Sex	Age	CD4/mm^3^	CD8/mm^3^	CD4/CD8	Cellularity Cells/mL	Macrophages	Lymphocytes	Neutrophils	Eosinophils	Hot Spot (HC × 40)	HC Location	Cluster Pattern
**1**	M	41	65	1520	0.04	100,000	60%	8%	32%	rare	165	IC + EC	III
**2**	M	52	15	293	0.05	88,000	85%	5%	10%	rare	55	IC + EC	I
**3**	M	40	150	629	0.23	114,000	88%	6%	5%	1%	95	IC + EC	II
**4**	M	43	20	79	0.25	79,000	88%	8%	4%	0%	122	IC + EC	II
**5**	F	46	11	1265	0.008	86,000	78%	12%	9%	1%	85	IC + EC	II
**6**	F	31	30	402	0.07	76,000	75%	15%	8%	2%	78	IC + EC	II
**7**	M	52	40	428	0.09	125,000	77%	13%	10%	0%	115	IC + EC	II
**8**	F	37	28	153	0.16	92,000	80%	12%	8%	0%	88	IC + EC	II
**9**	M	25	8	796	0.01	83,000	78%	10%	12%	rare	35	IC + EC	I
**10**	F	58	119	202	0.58	96,000	82%	10%	7%	1%	375	IC + EC	III
**11**	F	52	29	260	0.11	80,000	75%	15%	10%	0%	262	IC + EC	III
**12**	M	45	227	1455	0.15	100,000	85%	11%	4%	rare	9	IC	I
**13**	M	65	281	746	0.37	90,000	85%	8%	7%	0%	25	IC	I
**14**	M	38	6	157	0.03	120,000	86%	12%	2%	rare	132	IC + EC	II
**15**	M	55	Non-HIV/renal transplantation			160,000	59%	18%	23%	rare	66	IC	II
**16**	M	36	Non-HIV			117,000	73%	18%	9%	1%	10	IC	I
**17**	M	62	Non-HIV			138,000	82%	12%	6%	0%	4	IC	I

## Data Availability

Data of HIV patients being sensitive, upon reasonable request, and Commission Nationale Informatique et Libertés’ approval that the data may be shared.
